# The Role of Anorexia in Resistance and Tolerance to Infections in *Drosophila*

**DOI:** 10.1371/journal.pbio.1000150

**Published:** 2009-07-14

**Authors:** Janelle S. Ayres, David S. Schneider

**Affiliations:** Department of Microbiology and Immunology, Stanford University, Stanford, California, United States of America; University of Georgia, United States of America

## Abstract

Most infections induce anorexia but its function, if any, remains unclear. Because this response is common among animals, we hypothesized that infection-induced diet restriction might be an adaptive trait that modulates the host's ability to fight infection. Two defense strategies protect hosts against infections: resistance, which is the ability to control pathogen levels, and tolerance, which helps the host endure infection-induced pathology. Here we show that infected fruit flies become anorexic and that diet restriction alters defenses, increasing the fly's tolerance to *Salmonella typhimurium* infections while decreasing resistance to *Listeria monocytogenes*. This suggests that attempts to extend lifespan through diet restriction or the manipulation of pathways mimicking this process will have complicated effects on a host's ability to fight infections.

## Abstract 2

Infections initiate a signaling loop in which sick animals become anorexic, and the resulting change in diet alters the body's ability to fight infections in good and bad ways.

## Author Summary

Two routes to decreasing susceptibility to infection are resistance (the ability to clear pathogens) and tolerance (the ability to limit damage in response to pathogens). Anorexia induced by sickness puts animals into a diet-restricted state, a state that is generally believed to extend lifespan. We asked whether anorexia induced by sickness would alter the immune response. We measured the effects of diet restriction on both resistance and tolerance to two different infections in the fruit fly, *Drosophila melanogaster*. In one case we found that infection induced anorexia and the resulting diet restriction increased tolerance to this infection, thereby increasing survival of flies infected with this pathogen; however, this is not a universal effect. In a second case we found another pathogen that induced anorexia but here diet restriction lead to a reduction in resistance that collapsed the immune response and caused the fly to die faster. The relationship between diet restriction and immunity is complicated and must be evaluated on a pathogen-by-pathogen basis.

## Introduction

Infectious diseases are predicted to drive the natural selection of behaviors that increase fitness. Loss of appetite (anorexia) is a common behavior that sick animals exhibit when faced with an immune challenge [1]–[5]. Traditionally, anorexia was thought of as an adverse secondary response to infection that served no function to the host; immune responses are energetically expensive and thus an infection-induced reduction in food intake seems paradoxical [1]–[3]. Since this phenomenon occurs in so many animals, including both vertebrates and invertebrates, an alternative explanation is that this response is a conserved adaptive strategy to increase the chance of surviving an infection [1]–[3]. Experimental evidence from anorexia and acute starvation studies are consistent with this notion and suggest that this behavioral change is actively induced by the host during infection and is advantageous [3],[6]–[9]. For example, mice infected with *L. monocytogenes* (a firmicute and facultative intracellular pathogen) that were fed ad libitum had increased survival when compared to similarly infected but force fed mice [6]. The mechanism behind these changes in survival is unknown.

Diet restriction is a common method used for increasing an animal's lifespan. One explanation for this is that diet restriction increases responses required to survive stress [10]. Much progress has been made in determining the signaling pathways that trigger this process but the effector mechanisms remain elusive [10]. Most diet restriction experiments are done in the lab and a side effect of this is that the tested organisms are not exposed to a normal range of pathogens; thus, we do not have a deep understanding about how diet restriction can affect immune defenses. Individual immunological indicators of a potential immune response often improve upon diet restriction [1],[4],[11]. However, in the few cases where diet restricted animals have been given an infectious challenge, the diet restricted host often fared poorly, in spite of molecular indicators that its immune system would prevail [12]–[16]. Thus there seems to be a disconnect between the potential and realized immune response in diet restricted animals, suggesting that we are not measuring the relevant parts of the immune response.

Hosts can evolve two ways of defending themselves against infections [17]–[19]. The first, resistance, is the ability of the host to reduce pathogen levels. The second, tolerance, is the ability to limit the impact of infections. The theoretical basis for this distinction is grounded on work in plants, but recent work, from a number of groups, demonstrated that animals can also vary in their tolerance. In animals, tolerance traits appear relatively common and simple to identify genetically [17],[20]–[22]. For example, when screening for mutant flies with altered sensitivity to *L. monocytogenes*, we found that one-third of the mutants we recovered had no apparent defects in resistance and succumbed to infection because of defects in tolerance. At least when studying *Drosophila*, it seems clear that much has been missed in our studies of immunity by focusing on resistance mechanisms and ignoring tolerance [22].

Our work here was provoked by our identification of a mutation in a fly gustatory receptor, gr28b, that altered immune defenses [22]. We found that gr28b mutant flies had reduced appetites. This led us to the hypothesis that the feeding changes induced by anorexia might alter the immune response in an adaptive manner. We found that *L. monocytogenes* and *S. typhimurium* (a gamma proteobacterium and intracellular pathogen), both induce anorexia in infected flies, suggesting that diet restriction can be a normal part of the fly's response to infection. Mimicking this diet restriction by testing the gr28b mutant or by feeding the flies diluted food, we found that diet restriction reduced resistance against *L. monocytogenes* but increased tolerance against *S. typhimurium*. We propose that the degree of anorexia that is exhibited by an infected fly and the changes this decrease in food consumption imposes on the immune response will be continuously shaped by the pathogens a fly encounters in the wild.

The innate defenses important for resistance can be divided in three: the humoral, cellular, and melanization responses [23],[24]. The humoral response is the most deeply characterized and involves the secretion of antimicrobial peptides into the hemolymph (circulating “blood”) of the fly. Antimicrobial peptide transcript levels are regulated by the Toll and Imd pattern recognition pathways and these peptides are secreted predominantly by the fat body into the circulation of the fly and kill invading microbes [23]. Flies with mutations blocking the activation of these pathways quickly succumb to infections and have higher bacterial loads than do wild-type flies, suggesting that the principal defect in these mutant flies is in resistance [24]–[30]. The humoral response is induced over the course of several hours following a systemic infection. The cellular response is an immediate acting response and involves hemocytes (fly “blood” cells), which phagocytose small particles, encapsulate large particles, and secrete antimicrobial compounds [23],[24]. Melanization is a second immediate immune response in the fly occurring at sites of tissue damage and infection. Melanin deposits are visible as dark brown patches at these sites and its synthesis requires the proteolytic activation of the enzyme phenoloxidase. Reactive oxygen species are produced as a byproduct of this response that can cause damage to the fly thus affecting tolerance in addition to resistance [23],[24].

We examined the three arms of the fly immune response for changes caused by anorexia and diet restriction and found that melanization drops drastically upon diet restriction; the pattern of antimicrobial peptides induced during infection changes; but there were no apparent change in phagocytosis. The changes in melanization alone can explain the loss of resistance to *L. monocytogenes*. The explanation for the increase in tolerance to *S. typhimurium* is more complicated because the loss of melanization is expected to decrease resistance. We suggest that the fly compensates with another resistance mechanism, possibly antimicrobial peptides, while at the same time increasing tolerance. This work suggests that diet restriction will have complicated effects on immune defenses as it can alter both resistance and tolerance and its effects are microbe specific. This work supports the idea that the environment does not just affect a fly's immune response but rather is an integral part of immunity.

## Results/Discussion

### Infection-Induced Anorexia in *Drosophila*

**Figure 1:**
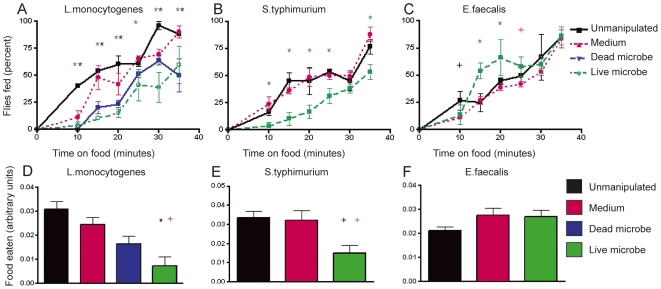
Effect of infection on appetite. Flies were infected with live or heat killed *L. monocytogenes*, live *S. typhimurium*, live *E. faecalis*, medium as a control, or left unmanipulated. Feeding was monitored by measuring the rate that flies took a meal (A–C) and the volume that they consumed during this meal (D–F). Feeding rate measurements: (A) *L. monocytogenes* 24 h postinfection; (B) *S. typhimurium* 24 h postinfection; (C) *E. faecalis* 24 h postinfection. To measure the volume of food consumed, fed flies were homogenized and the absorbance of an added blue dye was measured 24 h postinfection. (D) *L. monocytogenes*, (E) *S. typhimurium*, (F) *E. faecalis*. Error bars indicate standard error of the mean. Significance for (A–C) was assessed using a Fisher's exact test. Green asterisks represent live bacteria significantly different compared to both unmanipulated and media-injected flies. Blue asterisks represent dead bacteria (*L. monocytogenes* only) significantly different than both unmanipulated and media-injected flies. Black cross represents live bacteria significantly different from unmanipulated flies only. Pink cross represents live bacteria significantly different from media-injected flies only. Actual *p*-values are listed in Tables S1, S2, S3. Statistical analysis for (D–F) was done using ANOVA and a Tukey post-test; black asterisk indicates *p*<0.01 with respect to both unmanipulated and media-injected flies, black cross indicates *p*<0.05 with respect to unmanipulated flies only, and pink cross indicates *p*<0.05 with respect to media-injected flies only.

We measured infection-induced feeding changes in adult *Drosophila* challenged with three different bacterial pathogens of humans and *Drosophila*, *L. monocytogenes*, *S. typhimurium*, and *Enterococcus faecalis* (a firmicute and extracellular fly pathogen) (Figure 1) [18],[23],[31]–[33]. We chose these microbes because they represent very different types of bacteria and cause well characterized lethal infections in the fly; lethal microbes let us measure both increases and decreases in survival rates whereas nonpathogens only allow us to measure decreases. Feeding rates were determined by measuring how quickly flies took a meal when presented with new food and by recording how much food they consumed during this meal. The feeding rate assays were used primarily to determine the appropriate time window to perform the less subjective consumption assays. *L. monocytogenes* and *S. typhimurium* infections reduced food intake in both assays compared to unmanipulated and media-injected controls. By contrast, we detected no effect of *E. faecalis* infection on either feeding assay, demonstrating that illness-induced anorexia occurs in the fly in a microbe dependent manner. Dead *L. monocytogenes* also induced anorexia, suggesting that a simple immune response and not an active infection is sufficient to reduce the fly's appetite (Figure 1; Tables S1, S2, S3). Together, these results demonstrate that flies may enter a state of diet restriction when infected.

### gr28b Mutants Are Constitutively Anorexic

**Figure 2:**
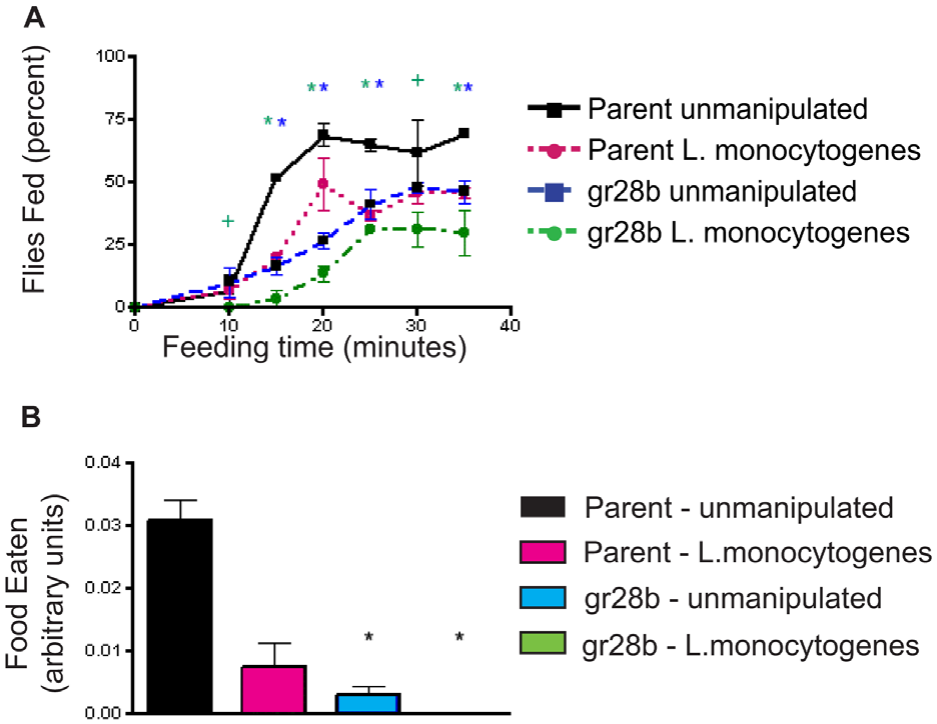
Effect of mutation of the gustatory receptor *gr28b* on appetite. Isogenic wild-type and *gr28b* mutants were assayed for feeding rates (A) and meal volumes (B). Error bars indicate standard error of the mean. Statistical analysis for (A) was done using a Fisher's exact test. Green asterisk indicates *Listeria*-infected gr28b mutants are significantly different than unmanipulated wild-type flies. Blue asterisks indicate that unmanipulated gr28b mutants are significantly different than unmanipulated wild-type flies. Green crosses indicate that *Listeria*-infected mutants are significantly different from unmanipulated wild-type flies only. Actual *p*-values are listed in Table S4. Statistical analysis for (B) was done using ANOVA and a Tukey post-test. Black asterisk indicates *p*<0.001 compared to unmanipulated wild-type flies.

We sought to determine how immune-induced diet restriction might alter the resistance and tolerance of the fly to a variety of pathogens. We previously identified a mutation in a taste receptor (gr28b) that reduced flies' resistance to *L. monocytogenes* infection while increasing defenses for *S. typhimurium* [22] and show here that these mutant flies eat less than wild-type controls (Figure 2; Table S4). We measured the feeding rates and ingestion volume of *gr28b* mutants and found that they ate at a significantly reduced rate compared to wild-type flies and that their ingestion volume was also reduced (Figure 2). These mutant flies also lived longer than parental controls when left unmanipulated (Figure S1) as would be expected for diet restricted flies [34]. Thus the gr28b mutant pointed to a potential functional link between anorexia and an altered immune response and provided a simple method of creating a constitutively anorexic fly.

### Anorexia and Diet Restriction Alter the Realized Immune Response

**Figure 3:**
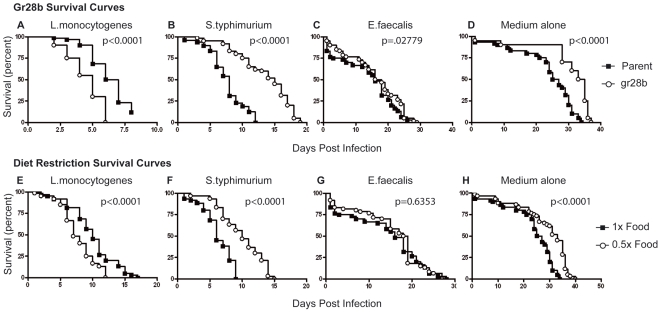
Effect of mutation of the gustatory receptor *gr28b* and diet restriction on sensitivity to infections. Isogenic wild-type and *gr28b* homozygous mutant flies were challenged with (A) *L. monocytogenes* (*p*<0.0001); (B) *S. typhimurium* (*p*<0.0001); (C) *E. faecalis* (*p* = 0.2779); or (D) medium alone (*p*<0.0001); and survival rates were measured and compared between flies given the two treatments. Wild-type flies fed on 1× and 0.5× diets, and were challenged with (E) *L. monocytogenes* (*p*<0.0001); (F) *S. typhimurium* (*p*<0.0001); (G) *E. faecalis* (*p* = 0.6053); or (H) medium alone (*p*<0.0001) and survival rates were measured. Significance was determined by log-rank test. Effects of *gr28b* mutations and diet restriction in unmanipulated flies on lifespan are shown in Figure S1.

To determine how anorexia affects the realized immune response of flies, we measured the survivorship of infected *gr28b* mutants and compared these rates to those of infected wild-type flies (Figure 3). Consistent with what we had observed previously [22], we found that when infected with *L. monocytogenes*, *gr28b* mutants died faster than wild-type flies; mutant flies died with a median time to death (MTD) of 4 d compared to 6–7 d for wild-type flies. In contrast, when infected with *S. typhimurium*, *gr28b* mutants lived longer than wild-type flies with a MTD of 15 d compared to 8 d. When infected with *E. faecalis*, *gr28b* and wild-type flies died at the same rate. Thus, gr28b flies have altered interactions with microbes but this could be due to the mutant's reduced appetite or to pleiotropic effects of the mutation. Anorexia is a symptom and there are potentially many different ways of becoming anorexic; thus we wanted to test another method that would simply restrict food intake.

To test the hypothesis that a reduction in food intake is responsible for the array of survival phenotypes we observe in *gr28b* mutant flies, we measured the effects of diet restriction on wild-type flies that we raised on standard diet that was diluted with 1% agar so that each stage of their lifecycle was completed on the diluted food. Typically in diet restriction studies, the introduction of restricted food occurs at the adult stage. We chose to utilize adult flies that had been raised their entire life on restricted food for two reasons: First, we reasoned that because gr28b mutants are inherently anorexic, they experience reduced food consumption at all stages of their life and we wanted to better emulate the reduced food intake of *gr28b* mutants. Second, for our initial experiments we used adult flies that had been diet restricted at 24 h prior to infection and we found that the phenotypes were enhanced as the amount of time on diet restricted food increased and we chose to maximize the effect. We infected food-restricted wild-type flies and compared survivorship to wild-type flies raised on standard food (Figure 3). Flies fed a 0.5× diet had phenotypes similar to the *gr28b* mutation in every way tested: diet restricted flies were more sensitive to *L. monocytogenes*; less sensitive to *S. typhimurium*; and showed no change in sensitivity to *E. faecalis*. Our results are in agreement with past observations that diet restriction has no effect on the survival rate of *E. faecalis* infected wild-type flies [15]. Previous studies examining the effects of diet restriction in the fly have reported neutral or weak positive effects on fly survival for *Pseudomonas aeruginosa* (gamma-proteobacterium and extracellular pathogen). Libert et al. reported that diet restriction has no effect on survival when challenged with *P. aeruginosa*. The pathogen load was not measured in this study and thus it cannot be determined whether there were compensatory changes in resistance and tolerance [14]. Diet restriction was reported to have positive effects on survival of *P. aeruginosa*-infected flies in an age dependent manner, where an increase in survival was seen in flies 30 d old or older but not 20 d or younger. This result demonstrated that the life history of a fly is another important factor to consider when measuring the interactions between diet restriction and immunity [14]. Pathogen load was not determined in this study and thus it cannot be determined whether the changes in old flies were due to changes in resistance or tolerance.

The lifespan of unmanipulated flies raised on a 0.5× diet was extended, which is in agreement with what has been previously observed in diet restricted flies and similar to what is seen in *gr28b* mutants. As diet restriction produced a complete phenocopy of the mutant phenotypes, we concluded that *gr28b* influences fly immunity by regulating food intake.

### Resistance and Tolerance Are Affected by Anorexia

**Figure 4:**
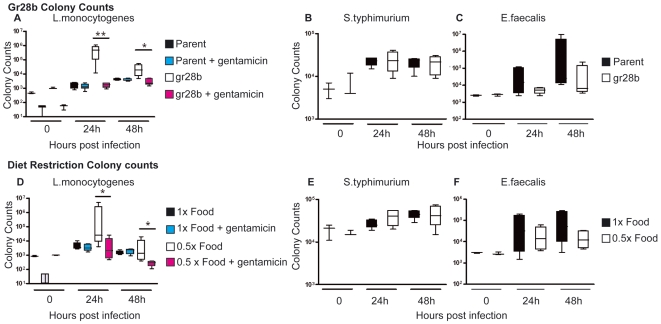
Effect of *gr28b* mutation and diet restriction on the growth of *L. monocytogenes* and *S. typhimurium*. Isogenic wild-type and *gr28b* homozygous mutant flies or wild-type flies fed on 1× and 0.5× diets were challenged with pathogens. Because *L. monocytogenes* infections showed a change in pathogen levels, half of the infected flies were injected with gentamicin to determine the relative abundance of intracellular and extracellular bacteria. Wild-type versus *gr28b* mutants: (A) *L. monocytogenes*; (B) *S. typhimurium*; and (C) *E. faecalis*. Regular food versus diet restriction: (D) *L. monocytogenes*; (E) *S. typhimurium*; (F) *E. faecalis*. Error bars indicate standard deviation. Statistical analysis was done using an unpaired two-tailed *t*-test. One asterisk indicates *p*<0.01 and two asterisks indicates *p*<0.005.

In *Drosophila*, we can determine whether a fly succumbs to an infection because of defects in resistance or tolerance mechanisms by monitoring both fly survival and pathogen growth over the course of the infection [12],[35]. We found that both resistance and tolerance mechanisms are affected by anorexia and the effect depended on the type of infection. Both *gr28b* mutant flies and diet-restricted wild-type flies exhibited increased growth of *L. monocytogenes* during infection (Figure 4). This growth, combined with the increased death rate we observed in both models, suggested that these flies died because of defects in resistance to *L. monocytogenes*; that is, reduced food intake blocked the ability of a fly to limit *L. monocytogenes* growth and thus the flies died faster. Because of the way we measure resistance and tolerance in the fly we cannot always measure changes in tolerance as microbe levels are changing; therefore, it is possible tolerance also changes under food restricted conditions in *L. monocytogenes*–infected flies. By contrast, during *S. typhimurium* infections, food restricted and *gr28b* mutants exhibited similar levels of bacteria to what we observed in wild-type flies (Figure 4) yet they lived longer. This suggested that resistance was unchanged but tolerance was increased.

**Figure 5:**
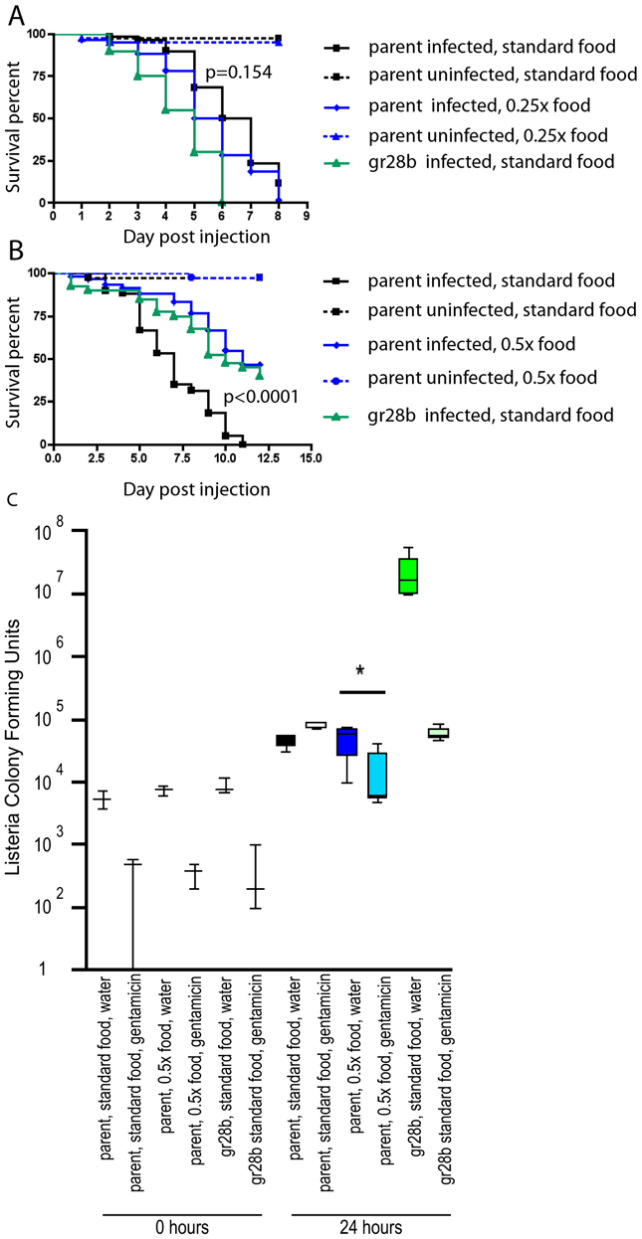
Effects of diet restriction on immunity when introduced post-eclosure. Five to 7-d-old adult males flies were collected and placed on a restricted diet or left at a 1× diet 24 h prior to infection. Survival of (A) *L. monocytogenes* (0.25× compared to 1×, *p* = 0.0154); (B) *S. typhimurium* (0.5× compared to 1×, *p*<0.0001); (C) growth of *L. monocytogenes*. Because *L. monocytogenes* infections showed a change in pathogen levels, half of the infected flies were injected with gentamicin to determine the relative abundance of intracellular and extracellular bacteria. Asterisk indicates *p* = 0.0173 as determined by an unpaired two-tailed *t*-test.

A drawback of our diet restriction protocol is that it raises the caveat that lifelong food limitation has effects on immunity because of developmental changes. To determine whether short-term diet restriction could produce symptoms similar to those seen in *gr28b* flies or flies diet restricted since hatching, we placed flies on diet restriction food 24 h before challenging them with microbes (Figure 5). *L. monocytogenes*–infected flies showed significantly decreased survival, whereas *S. typhimurium*-infected flies showed increased survival comparable to that seen in *gr28b* flies. These experiments support the idea that diet restriction in adults affects defenses by altering the fly's physiology without causing developmental changes.

In summary, diet restriction has varied effects on tolerance and resistance in the fly: diet restriction causes no change during *E. faecalis* infections, reduces resistance to *L. monocytogenes*, and increases tolerance to *S. typhimurium*.

### Anorexia Affects Multiple Arms of the *Drosophila* Innate Immune Response

To determine the mechanism behind the changes in resistance and tolerance we observe under diet restriction, we examined the three resistance mechanisms of the *Drosophila* innate immune response that are important for limiting microbial growth: phagocytosis, antimicrobial peptide production, and melanization [23],[24]. We saw no change in phagocytosis rates in anorexic flies (Figure S2) but found significant differences in the other two immune responses.

**Figure 6:**
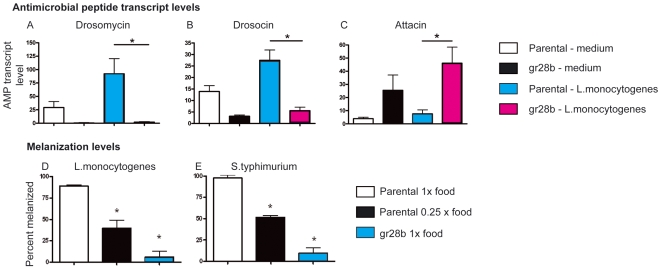
Effect of anorexia and diet restriction on antimicrobial peptide expression and melanization. *gr28b* mutants and diet restricted flies were injected with *L. monocytogenes*, and antimicrobial peptide transcript levels were monitored at 6 h postinfection by quantitative real-time reverse-transcription PCR. Transcript levels were recorded as the ratio of the antimicrobial peptide transcript divided by a housekeeping transcript (ribosomal protein 15a) and normalized to 1 for unmanipulated wild-type flies. (A) drosomycin; (B) drosocin; (C) attacin. Error bars report standard error of the mean. ANOVA and Tukey tests were performed for statistical analysis and asterisks indicate *p*<0.05. Melanized spots were recorded in (D) *L. monocytogenes* and (E) *S. typhimurium* infections. ANOVA and Tukey test were done for statistical analysis. Asterisks represent *p*<0.001.

We measured the levels of antimicrobial peptide transcript levels in *L. monocytogenes*–infected *gr28b* mutants and food-restricted flies and found that they elicit similar effects (Figure 6; unpublished data). In *gr28b* mutants we found that postchallenge transcript levels for drosomycin and drosocin were significantly reduced compared to wild-type flies (*gr28b*, 20×). However, anorexia did not affect all antimicrobial peptide transcripts in the same way; attacin transcripts were found at higher levels in *gr28b* flies (Figure 6), whereas diptericin transcripts showed no consistent change (unpublished data). The antimicrobial peptide response has been well-characterized for newly infected but otherwise healthy flies; We found that three antimicrobial peptides typically described as being coordinately regulated (attacin, diptericin, and drosocin) are regulated independently during diet restricted conditions. We show that the rules governing AMP expression are variable and depend not only upon the specific immune challenge but also upon environmental conditions.

To determine if diet restriction affected the melanization response, we infected flies with *L. monocytogenes* or *S. typhimurium*, both of which elicit a robust disseminated melanization response in the fly, and examined flies for evidence of melanization (Figure 6) [35]. We found that approximately 90% of our wild-type flies fed a standard diet exhibited melanization, whereas less than 10% of *gr28b* mutants melanized when infected with either *S. typhimurium* or *L. monocytogenes*. We also observed a significant reduction in melanization in diet restricted wild-type flies. Nutrient deprivation studies in the mosquito and the darkling beetle have also demonstrated that melanization is reduced under food restricted conditions [36],[37]. These results demonstrate that diet restriction/anorexia causes down-regulation of infection-induced melanization.

In wild-type flies, *L. monocytogenes* establishes an intracellular infection; in *CG3066* fly mutants defective in melanization, we find an extracellular population of bacteria in addition to the typical intracellular population [35]. Because diet restriction causes a reduction in the melanization response, we hypothesized that flies will also produce an extracellular population of *L. monocytogenes* when diet restricted. To test this idea we performed a gentamicin chase experiment (Figures 4 and 5) [32]. Infected flies were injected with the antibiotic gentamicin or with water at 0, 24, and 48 h postinfection and surviving bacteria were counted; gentamicin kills extracellular bacteria while the intracellular bacteria are protected. Indeed, *gr28b* flies and diet restricted flies had a large extracellular population of *L. monocytogenes*, in contrast to wild-type and normally fed flies, which did not. We also observe this effect in flies that were diet restricted only 24 h prior to infection but the phenotype is dramatically enhanced in flies that were raised on a restricted diet (Figures 4 and 5).

The effects of diet restriction on melanization seem easily interpretable with respect to *L. monocytogenes* infections but reveal an exciting complexity with *S. typhimurium*. Inhibition of melanization in a *CG3066* mutant has the same effect on *L. monocytogenes* and *S. typhimurium* infections— a loss of resistance [35]. Therefore, the loss of melanization in diet restricted flies can explain the entire *L. monocytogenes* infection phenotype because the phenotype is the same as that seen in *CG3066* mutants. This is not the case with *S. typhimurium*; loss of melanization was anticipated to reduce resistance to *S. typhimurium*; instead, we found an increase in tolerance and no change in resistance. If a resistance mechanism is lost when melanization is removed because of diet restriction, some resistance mechanism must replace it to prevent *S. typhimurium* growth. In addition, the increase in tolerance in these flies needs to be explained. One possible explanation is that, in diet restricted flies, the loss of melanization increases the tolerance to *S. typhimurium* infections and the rebalancing of antimicrobial peptide levels replaces the resistance that would have been lost through the loss of melanization. More complex explanations require proposing the induction of unknown resistance and tolerance mechanisms.

Regardless of the effects diet restriction has on individual resistance mechanisms, the practical outcome of this work is its demonstration that sensitivity to infections changes in diet restricted flies. This can benefit the host, as is seen in *S. typhimurium* infected flies or harm the host, as seen in *L. monocytogenes* infections. In the field, the effect of an anorexic response to infection on evolution should depend upon the pathogens to which a population was exposed. For example, *Salmonella*-like organisms should drive an increase in anorexia responses while *Listeria*-like pathogens would have the opposite effect.

By highlighting the contribution of feeding to defense, this work has practical implications for fly immunity experiments. Changes in nutrition due to food variation could explain week-to-week alterations in survival curves or plating experiments within a lab. Similarly, differences in food recipes could explain lab-to-lab variability. The finding that diet affects both specific antimicrobial peptide transcript levels and melanization means that experiments using these responses as outputs must be interpreted carefully; for example, when pathogens are fed to flies, test subjects fed a high dose of bacteria receive a different diet than flies fed food lacking these microbes and might be expected to have a different immune response just because the food differs. Microbe dose may be difficult to regulate when feeding sick flies if anorexia is induced by the infection; this could lead to confounding results where mutant flies that do not become anorexic take larger doses of the infecting microbes than do wild-type flies. In cases like this, nonanorexic flies could pay for their dietary indiscretion with their lives. Recent work in a variety of animals demonstrates that our native microbiota affect our immune system [38],[39]. Certainly some of this comes from the direct interaction between the microbes and pattern recognition pathways but native microbiota often play a role in contributing to host nutrition and metabolism. Therefore, care should be taken when comparing immune responses between axenic and normally raised flies; not all immune changes will be due to the mere physical exposure of the fly to microbes.

This work adds to a growing literature on regulatory loops linking the fly's immune responses to the environment [40]–[46]. A fly's susceptibility to infection is altered by temperature and several insects have behavioral fevers induced by infection; such fevers can affect the outcome of infections [47]–[52]. Immune challenges alter circadian rhythms in flies and this can feedback to change immunity in ways that can be either helpful or destructive [43],[45],[46]. It perhaps came as no surprise that nutrition affects fly immunity but what we demonstrated here was that during an immune response, the fly actively alters its nutrition and, again, this leads to feedback loops that can aid or collapse the immune response. Recent work on the African armyworm, *Spodoptera exempta*, demonstrates that this insect may not only change its appetite, but also changes its preference for protein or carbohydrate rich foods during infection [53]. This raises the possibility that the anorexia response we measure in flies could be complicated as it is difficult to distinguish an “I am not that hungry” response from “yuck, I do not want to eat this junk,” if the flies are presented with just one food choice. All of this suggests that the fly's immune response isn't merely sensitive to ambient environmental conditions, rather the fly uses the environment as an integral part of its immune response.

Diet restriction can increase the lifespan of animals allowed to come to a generic “natural death” in the lab [10]. Though the ultimate mechanisms regulating aging remain unknown, signaling pathways linking diet restriction and aging are emerging as potential drug targets. Our model provided an opportunity to measure the effects of a naturally induced diet restriction on deaths induced by different pathogens. The work reported here should raise a cautionary flag as it demonstrates that diet restriction can have complex effects on the realized immune response of a diet-restricted animal. We must determine how diet restriction affects realized immune responses in addition to basic immune effectors and anticipate that this will differ in a pathogen-specific manner.

## Methods

### Fly Strains

The wild-type parental strain used in all experiments is *white^1118^* (Bloomington stock center, stock 6326). The *gr28b^c01884^* allele was obtained from Bloomington stock center (stock 10743). The piggy bac line was generated on the *white^1118^* background and backcrossed further onto the *white^1118^* background for four generations. Flies were kept in standard fly bottles containing dextrose medium and raised under a 12-h light-dark cycle at 25°C prior to experiments.

### Bacterial Strains

*L. monocytogenes* strain 10403s [54] was stored at −80°C in brain-heart infusion (BHI) broth containing 15% glycerol. *S. typhimurium* strain SL1344 and *E. faecalis* strain V583 were stored at −80°C in Luria Bertani (LB) medium containing 15% glycerol.

### Pathogen Culture Conditions

*E. faecalis* and *S. typhimurium* cultures were grown overnight at 37°C in LB medium. *E. faecalis* cultures were shaken, while *S. typhimurium* cultures were grown standing. *S. typhimurium* cultures were diluted to OD_600_ of 0.1 with fresh LB medium prior to injection. *E. faecalis* cultures were diluted to an OD_600_ of 0.05 with medium. *L. monocytogenes* was grown overnight in BHI medium. *L. monocytogenes* was grown standing and injected at an OD_600_ of 0.01.

### Injections

5- to 7-d-old males were used for injection. Flies were anesthetized with CO_2_ and injected with 50 nl of culture or medium using a picospritzer (Parker Hannifin) and pulled glass needle. Flies were injected in the anterior abdomen on the ventrolateral surface. Flies were then placed in vials containing dextrose medium in groups of 20 (or ten for feeding assays) and incubated at 29°C 65% CO_2_ under a 12-h light-dark cycle. Flies were injected with 1,000 CFUs of live or dead *L. monocytogenes*, 10,000 CFUs of *S. typhimurium*, or 5,000 CFUs of *E. faecalis*

### Survival Curves

For each microbe tested, *w^1118^* and *gr28b* mutants were injected with the microbe or medium as a control. Flies were placed in dextrose vials in groups of 20 after injection and a total of 60 flies were assayed for each condition. The number of dead flies was counted daily. Using Prism software, Kaplan-Meier survival curves were generated and statistical analysis was done using log-rank analysis. Survival was tested for each microbe at least three times and gave similar results for each trial. All survival experiments were done at 29°C.

### CFU Determination and Gentamicin Chase

Infected flies were homogenized in media supplemented with 1% Triton X-100 and serially diluted. Dilutions were plated on LB agar plates and incubated over night. The data were plotted as box and whiskers plots using Graphpad Prism software for three independent experiments. Using an unpaired two-tailed *t*-test, the *p*-value was determined. For the gentamicin chase experiments, flies were injected with 50 nl of 1 mg/ml gentamicin or water 3 h prior to homogenizing and plating. Flies were incubated at 29°C post infection

### Quantitation of Antimicrobial Peptide Transcripts

Total RNA was extracted from infected or control flies that were incubated at 29°C in groups of five flies using the Qiagen RNeasy kit (Qiagen) at 0 and 6 h postinjection. The samples were treated with DNase (Promega). Quantitative real-time RT-PCR was performed with rTth polymerase (Applied Biosystems) using a Bio-Rad icycler (Bio-Rad) and the following primer sets: drosomycin 5′ 5′-gacttgttcgccctcttcg-3′, drosomycin 3′ 5′-cttgcacacacgacgacag-3′, drosomycin Taqman probe 5′-tccggaagatacaagggtccctgtg-3′, diptericin 5′ 5′-accgcagtacccactcaatc-3′, diptericin 3′ 5′-cccaagtgctgtccatatcc-3′, diptericin taqman 5′-cagtccagggtcaccagaaggtgtg-3′, attacin 5′ 5′-caatggcagacacaatctgg-3′, attacin 3′ 5′-attcctgggaagttgctgtg-3′, attacin Taqman probe 5′-aatggtttcgagttccagcggaatg-3′, drosocin 5′ 5′-ttcaccatcgttttcctgct-3′, drosocin 3′ 5′-agcttgagccaggtgatcct-3′, drosocin Taqman probe 5′-gtttttgccatggctgtggccact-3′. Concentrations of AMP transcripts were normalized to the expression of the *Drosophila* ribosomal protein 15a transcript for each sample [55]. All experiments were performed with three biological replicates and each experiment was performed at least three times.

### Melanization Assay

Flies were infected as described above with *L. monocytogenes* or *S. typhimurium* and incubated at 29°C for 4 d. Flies were then visualized by light microscopy and examined for a disseminated melanization response. Flies that exhibited melanization beyond what is observed at the injection site are scored as positive for a melanization response. Flies that observe no melanization or melanization only at the site of injection are scored as negative for a melanization response.

### Diet Restriction Assays

All experiments were performed as described above using flies that were raised on restricted diets. Restricted food was generated by diluting the standard 1× diet 1∶2 or 1∶4 in 1% agar water to generate the 0.5× or 0.25× diet. Vials were placed on a rocker while food solidified to prevent settling of the food.

### Feeding Assays

Feeding rate and ingestion amount were done using standard fly dextrose diet supplemented with 0.1% bromophenol blue and 0.5% xylene cyanol [56],[57]. Our standard fly food recipe contains the following chemicals in 1 l of cooked food: 129.4 g dextrose, 7.4 g agar, 61.2 g corn meal, 32.4 g yeast, 2.7 g tegosept. Flies were injected as described above or were left unmanipulated and were incubated at 29°C under 12-h light-dark cycle for at least 24 h to allow flies adequate time to recover from CO_2_ treatment on their standard diet without tracking dye. To measure feeding rate, flies were transferred to vials containing food with tracking dye and incubated at room temperature for time point collections. We chose to keep the flies at room temperature because we found that the opening and closing of the incubator door at each time point disturbed feeding activity. Experiments were performed at the same time of day (2 pm, ZT5). At each time point flies are then transferred to empty vials that contain no food. At the end of the time course all flies are examined for the presence of blue dye inside their bodies and the percentage of flies that ingested a meal was recorded. For each experimental condition three groups of at least ten flies were tested for each time point. The average percentage of flies that ingested a meal was plotted and a Fisher's exact test was done for statistical analysis.

To measure ingestion amount at the desired time points at least three groups of ten flies that have been feeding on the tracking food were collected and homogenized in 100 µl of 1×TE buffer with 0.1% Triton X-100. 1 ml of 1×TE was added and then homogenates were centrifuged at 14,000 rpm for 3 min. Supernatants were collected and the absorbance at 614 nm was measured. The average absorbance for each experimental condition was recorded and ANOVA and a Tukey test was done for statistical analysis.

## Supporting Information

Figure S1 ***gr28b*** **mutants and diet restricted flies have extended lifespans.** Five to 7-d-old male flies were placed on standard dextrose diet or on 0.5× concentrated dextrose diet and incubated at 29°C. The number of dead flies was counted daily until all flies were dead. Survival curves and median time to death are presented. Survival rates were analyzed by log-rank analysis.
(0.53 MB EPS)

Figure S2 **The cellular response in** ***gr28b*** **mutants is unchanged.** Wild-type and *gr28b* mutants were injected with FITC-labeled *Escherichia coli* and *S. aureus* and incubated at room temperature for 1 h to allow phagocytosis to occur. Flies were then injected with trypan blue, which quenches any extracellular fluorescence but intracellular bacteria are protected from the quenching agent. Flies were visualized by fluorescent microscopy to look for differences in fluorescence levels.
(4.24 MB EPS)

Table S1 ***p*** **-Values for rate of feeding in** ***L. monocytogenes*** **infected flies.** Flies were infected with live or heat killed *L. monocytogenes*, medium as a control or left unmanipulated. Flies were placed on food supplemented with a blue dye and the rates of feeding were monitored. A Fisher's exact test to determine the significance at each time point was done and the *p*-values are reported here.
(0.01 MB XLS)

Table S3 ***p*** **-Values for the rate of feeding of** ***E. faecalis*** **infected flies.** Flies were infected with live *E. faecalis*, medium as a control, or left unmanipulated. Feeding was monitored by measuring the rate that flies took a meal. A Fisher's exact test to determine the significance at each time point was done and the *p*-values are reported here.
(0.01 MB XLS)

Table S2 ***p*** **-Values for rate of feeding in** ***S. typhimurium*** **infected flies.** Flies were infected with live *S. typhimurium*, medium as a control, or left unmanipulated. Feeding was monitored by measuring the rate that flies took a meal. A Fisher's exact test to determine the significance at each time point was done and the *p*-values are reported here.
(0.01 MB XLS)

Table S4 ***p*** **-Values for the rate of feeding of unmanipulated and** ***L. monocytogenes*** **infected gr28b mutants.** Flies were infected with live *L. monocytogenes*, or left unmanipulated. Feeding was monitored by measuring the rate that flies took a meal. A Fisher's exact test to determine the significance at each time point was done and the *p*-values are reported here.
(0.01 MB XLS)
